# Bile-volatile organic compounds in the diagnostics of pancreatic cancer and biliary obstruction: A prospective proof-of-concept study

**DOI:** 10.3389/fonc.2022.918539

**Published:** 2022-11-21

**Authors:** Ville Teränen, Samuli Nissinen, Antti Roine, Anne Antila, Antti Siiki, Yrjö Vaalavuo, Pekka Kumpulainen, Niku Oksala, Johanna Laukkarinen

**Affiliations:** ^1^ Department of Gastroenterology and Alimentary Tract Surgery, Tampere University Hospital, Tampere, Finland; ^2^ Faculty of Medicine and Health Technology, Tampere University, Tampere, Finland; ^3^ Department of Internal Medicine, School of Medicine, University of Eastern Finland, Kuopio, Finland

**Keywords:** VOC, pancreatic cancer, biliary obstruction, FAIMS, bile, ERCP, pancreatitis, cholangiocarcinoma

## Abstract

**Objectives:**

Detection of volatile organic compounds (VOCs) from bodily fluids with field asymmetric waveform ion mobility spectrometry (FAIMS) and related methods has been studied in various settings. Preliminary results suggest that it is possible to detect prostate, colorectal, ovarian and pancreatic cancer from urine samples. In this study, our primary aim was to differentiate pancreatic cancer from pancreatitis and benign tumours of the pancreas by using bile samples obtained during endoscopic retrograde cholangiopancreatography (ERCP). Secondarily, we aimed to differentiate all pancreatic region malignancies from all other kinds of benign causes of biliary obstruction.

**Methods:**

A bile sample was successfully aspirated from 94 patients during ERCP in Tampere University Hospital. Hospital and patient records were prospectively followed up for at least two years after ERCP. Bile samples were analysed using a Lonestar chemical analyser (Owlstone, UK) using an ATLAS sampling system and a split-flow box. Diagnoses and corresponding data from the analyses were matched and divided into two subcategories for comparison. Statistical analysis was performed using linear discriminant analysis, support vector machines, and 5-fold cross-validation.

**Results:**

Pancreatic cancers (n=8) were differentiated from benign pancreatic lesions (n=9) with a sensitivity of 100%, specificity of 77.8%, and correct rate of 88%. All pancreatic region cancers (n=19) were differentiated from all other kinds of benign causes of biliary obstruction (n=75) with corresponding values of 21.1%, 94.7%, and 80.7%. The sample size was too small to try to differentiate pancreatic cancers from adjacent cancers.

**Conclusion:**

Analysing bile VOCs using FAIMS shows promising capability in detecting pancreatic cancer and other cancers in the pancreatic area.

## Introduction

Detection of volatile organic compounds (VOCs) from bodily fluids with field asymmetric waveform ion mobility spectrometry (FAIMS) and similar methods has demonstrated extensive capabilities in VOC analyses. Promising results have been reported in the detection from urine of pancreatic cancer, prostate cancer, bladder tumours, colorectal cancer, ovarian cancer, non-alcoholic fatty liver disease and urinary tract infection pathogens (1–11). Exhaled breath has been used for the detection of pancreatic, breast, lung and colorectal cancer as well as COVID-19 (12, 13). Moreover, faecal VOCs have been studied in the context of inflammatory bowel disease, colorectal cancer and infectious diarrhoea (14–16). Methods based on the same principles have also been used in the intraoperative analysis of diathermy smoke to detect cancerous tissue on resection margins (17, 18).

Pancreatic cancer diagnostics is a notoriously challenging topic as the majority of cases are detected at either a locally advanced or metastatic stage of the disease. Nowadays the diagnosis is mainly based on high radiation dose computed tomography. However, when a pancreatic mass is detected in an imaging test, it may be challenging to assess if the finding is malignant or benign as radiological findings of both aetiologies can mimic each other (19). This may complicate the clinical decision-making whether to swiftly resect the mass or to opt for alternative modalities and tissue sampling. Either way, a significant proportion of patients with pancreatic cancer or other biliary obstruction require preoperative biliary drainage, which is often performed in endoscopic retrograde cholangiopancreatography (ERCP). This procedure also enables gathering brushings and biopsies in the pancreatic region for diagnostic aid, but their sensitivity in reaching a definitive diagnosis remains low (20, 21). Currently, endoscopic ultrasound fine-needle aspiration (EUS-FNA) is the gold standard for obtaining pancreatic tissues (22, 23). Furthermore, diagnostic ERCP has a potential risk of pancreatitis. Regardless, analysis of bile VOCs could offer new opportunities for the diagnostic issue. So far, only one study (24) has been presented on using bile VOCs for differentiating between pancreatic cancer and chronic pancreatitis.

Our primary aim in this study was to differentiate malignant and benign causes of biliary obstruction using a bile sample obtained in an ERCP. Firstly, our aim was to differentiate pancreatic adenocarcinoma from all kinds of benign lesions of the pancreas. Secondly, we aimed to differentiate all pancreatic region malignancies from all kinds of benign causes of biliary obstruction.

## Materials and methods

### Data collection

One hundred and nine patients undergoing ERCP for any reason in Tampere University Hospital (TAUH) between October 2017 and January 2019 were recruited. Ethical approval was obtained from TAUH (R17072) and the patients gave written consent. A bile sample of 1-2 millilitres was successfully aspirated from the bile duct during ERCP from 94 patients. Bile samples in separate cuvettes were immediately frozen to -20°C in TAUH, from which they were transferred to an ultra-low temperature freezer (-70°C) in a laboratory of Tampere University.

**Figure 1:**
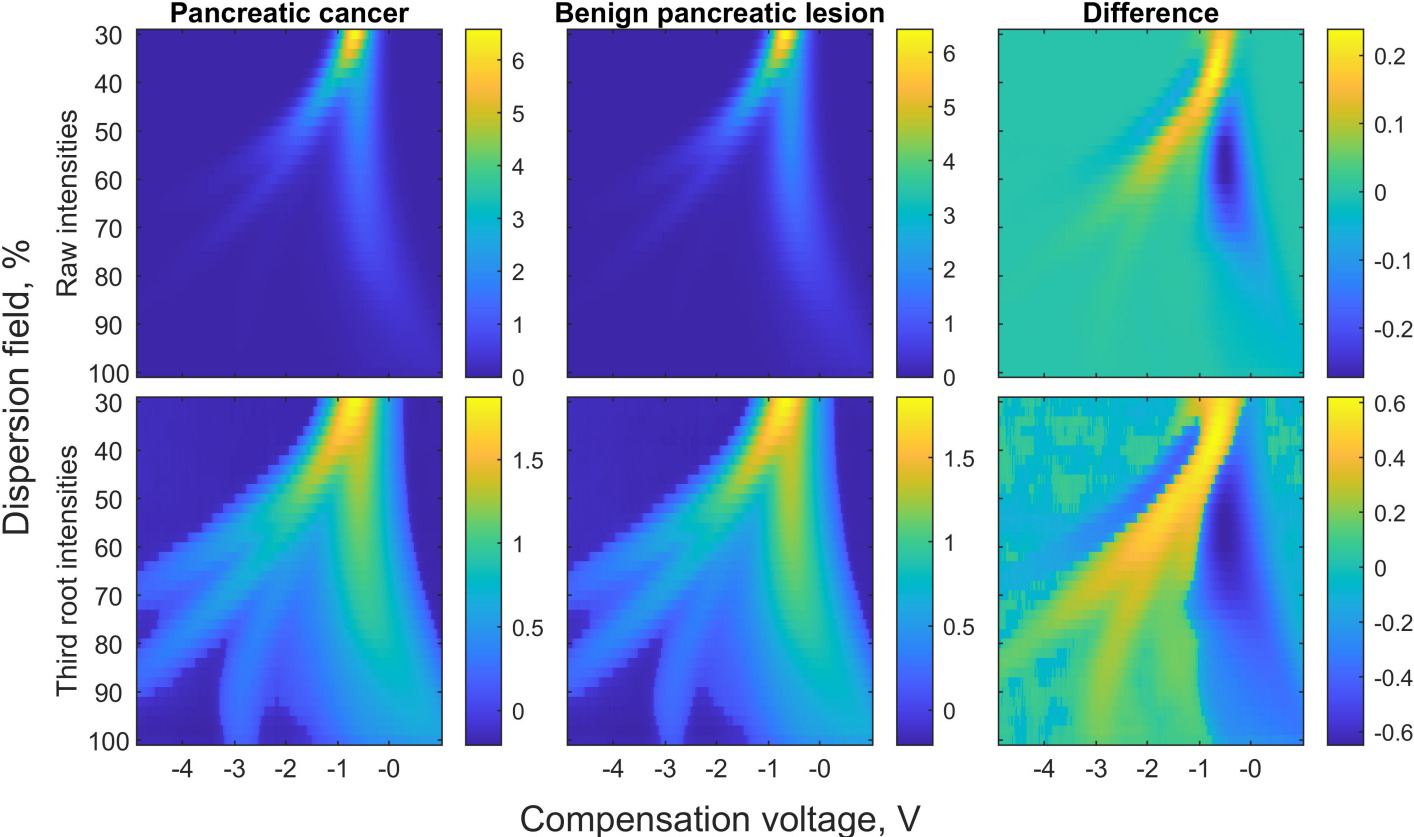
FAIMS spectra of bile in pancreatic cancer and benign pancreatic lesion groups and their differences. In the first row, water molecules bend far left in the electric field, while indistinguishable molecules composing the background spike bend to the far right. Discriminative molecules are found between these two. In the second row, the differences are accentuated by calculating third roots.

All samples were analysed in January 2019 on four consecutive days using Lonestar chemical analyser (Owlstone, UK), employing an ATLAS sampling system and a split-flow box. Samples were warmed up to room temperature prior to analysis. Samples were diluted by pipetting 0.5 millilitres of bile and adding 4.5 millilitres of sterilized water. Flow settings MFC1 = 500, MCF=200 and MFC3 = 2200 were used. Each sample took 120 seconds to scan. We ran two scans with sterilized water between each bile sample for cleaning purposes. Each sample was measured in 26,112 points, at 51 values of the dispersion field (DF) from 0% to 100%, and 512 values of the compensation voltage (CV) from -6.0 V to 5.9937 V. Parts of the measurement space, containing only noise, were removed to focus on the relevant information (**Figure 1**). The selected data contains 9036 points per sample, 36 DF values from 30% to 100%, and 251 values of CV from -4.3805 V to 1.4873 V. The FAIMS system was left in continuous scanning mode with a sterilized water sample every night to reduce carry-over effects. Positive mode compensation voltage sweep curve representing background smell was aimed at under 35%.

Patient records were followed up until August 2021. Data on patients’ age, sex, primary diagnosis at the time of the procedure, specified diagnosis and possible death during the follow-up period were gathered. The specified diagnosis was obtained by re-evaluating the primary diagnosis at the time of ERCP against possible subsequent findings and events. The diagnosis to be used in statistical analysis was updated, for example if the primary diagnosis was nonspecific and the patient was diagnosed with cancer shortly after the ERCP. The diagnosis was also updated if another cancer was discovered and had likely already been present at the time of ERCP, as even more distant cancers are known to affect bodily fluid smell profiles, as discussed below. The diagnosis changed from benign to malignant in ten patients during the follow-up period. In nine out of those ten patients, the more specific diagnoses also affected the final analysis. More detailed information about adjusted diagnoses is presented in **Table 1**. Conclusive diagnoses were sorted into 11 categories presented in detail in **Table 2**.

**Table 1:** Pathology categorizations changed in nine patients during the follow-up.

Patient	Primary diagnosis	Category of primary diagnosis	Exact diagnosis used in the analysis	Category of conclusive diagnosis	Follow-up time needed to acquire the final diagnosis
I	Stricture of common bile duct and lesion of the ampulla of Vater not otherwise specified	4	Melanoma and benign tumour of the pancreas not otherwise specified	11	34 months
II	Choledocholithiasis and tumour in the ampulla of Vater, low-grade dysplasia in brush cytology	4	Pancreatic adenocarcinoma with liver metastases	8	12 months
III	Stricture of bile duct not otherwise specified	4	Pancreatic adenocarcinoma	8	1 month
IV	Stricture of the bile duct, tubulovillous adenoma of ampulla of Vater, low-grade dysplasia in brush cytology	4	Ampullary cancer	10	8 months
V	Stricture of bile duct not otherwise specified	4	Cholangiocarcinoma with extensive invasion into the duodenum wall	9	6 months
VI	Radiologically suspected cholangiocarcinoma, low-grade dysplasia in brush cytology	9	Colorectal adenocarcinoma with extensive metastases	11	1 month
VII	Stricture of bile duct not otherwise specified	4	Ampullary cancer	10	1 month
VIII	Stricture of bile duct not otherwise specified	4	Pancreatic adenocarcinoma	8	14 months
IX	Tumour of the pancreas not otherwise specified	4/5	Pancreatic adenocarcinoma	8	1 month

Numbers corresponding to categories: 1: primary sclerosing cholangitis, 2: cholangitis or cholecystitis with or without cholelithiasis or choledocholithiasis, 3: cholelithiasis or choledocholithiasis without inflammation, 4: benign cause: stricture, adenoma etc., 5: benign cause: intrapapillary mucinous neoplasm or other cystic tumour, 6: acute pancreatitis, 7: chronic pancreatitis, 8: pancreatic adenocarcinoma, 9: cholangiocarcinoma, 10: duodenal carcinoma, 11: other cancers and their metastases.

**Table 2:** Pathology categorizations used in the final analysis in all patients.

	No. of patients (N = 94) and percentage
**All benign causes of** biliary obstruction **n = 75**	
Primary sclerosing cholangitis	22 (23.4%)
Cholangitis or cholecystitis with or without cholelithiasis or choledocholithiasis	11 (11.7%)
Cholelithiasis or choledocholithiasis without inflammation	21 (22.3%)
Benign cause: stricture, adenoma etc.	12 (12.8%)
Benign cause: intrapapillary mucinous neoplasm or other cystic tumor	0
Acute pancreatitis	6 (6.4%)
Chronic pancreatitis	3 (3.2%)
**All malignancies n = 19**	
Pancreatic adenocarcinoma	8 (8.5%)
Cholangiocarcinoma	6 (6.4%)
Other cancers or their metastases	3 (3.2%)
Duodenal cancer	2 (2.1%)

### Statistical analysis

Data were analysed by a statistician using MATLAB R2021b (MathWorks Inc, Natick, MA, USA). FAIMS data were used to train classification methods in order to meet the objectives of this study. Linear discriminant analysis (LDA) and support-vector machine (SVM) were utilised for classification (25, 26). Both methods were cross-validated with leave-one-out (LOO) and 5-fold cross-validations (27, 28) and the data were weighted (with the inverse class size) to compensate for the imbalance in the number of patients in the classes. In cross validation a subset of the data is left out as a test set and the classifiers are identified using the rest of the data. In LOO one data point at a time is left out as a test data and in 5-fold one the data set is devided into 5 groups and one group at a time is left as test data. The results are classifiations of the test sets. The FAIMS (spectral images) were smoothed with 3 by 15 sliding window to reduce local measurement noise. The best discriminating locations (pixels in the spectral image) of the data space were identified by two-sided Wilcoxon rank-sum test. The most significant pixels (Bonferroni corrected p-value below 0.05) were selected for classification.

In our primary research question, benign pancreatic lesions were taken to be acute pancreatitis, chronic pancreatitis and benign cystic lesions (such as intraductal papillary mucinous neoplasm; IPMN), while pancreatic cancer was explicitly defined as pancreatic adenocarcinoma. In our secondary research question, primary sclerosing cholangitis, cholangitis, cholecystitis, gallstones, biliary duct stones, benign strictures, adenomas, pancreatic cystic lesions, acute and chronic pancreatitis were taken to be benign causes of biliary obstruction. In our research material, all malignant diagnoses consisted of pancreatic adenocarcinomas, cholangiocarcinoma, duodenal carcinomas and other cancerous metastases in the pancreatic region. Pathology categorizations used in the final analysis are listed in **Table 2**.

## Results

The study population consisted of 109 patients with a median age of 64 years (range of 16 - 91 years), and 56% of them were men. Of the 94 bile samples collected, in 17 the indication for ERCP was a known PSC, while the remainder underwent ERCP because of acute biliary obstruction. Details of demographics, sample sizes, follow-up times and altered primary diagnoses are presented in **Tables 1**–**3**.

**Table 3:** Demographics, sample sizes and follow up times in all patients.

	No. of patients (N=94) and percentage or median and range
Age (median, range)	64 years (18-91)
Sex (male)	51 (54.3%)
Follow-up time (median, range)	40 months (31-46)
Deaths during follow-up	17 (18.1%)
Age at death (median, range)	70.5 years (44-90)
Lifetime after ERCP (median, range)	8 months (1-39)
Diagnosis changed during follow-up	15 (16.0%)
Diagnosis changed from benign to malignant cause	10 (10.6%)
Diagnosis change led to change in diagnosis used in the analysis	9 (9.6%)
Time until diagnosis changed (median, range)	3 months (4 days - 33.8 months)

ERCP, endoscopic retrograde cholangiopancreatography.

LDA classification model with 5-fold cross-validation was the most accurate statistical method in the differentiation of pancreatic lesions. Pancreatic adenocarcinomas (n = 8) were differentiated from benign pancreatic lesions (n = 9) with a sensitivity of 100%, specificity of 77.8% and correct rate of 88.2%. Data suggesting a present malignancy acquired by FAIMS could have improved the outcomes in half of pancreatic cancer patients. Differences in FAIMS spectra of pancreatic cancer and benign pancreatic lesion samples are presented in **Figure 1**.

SVM classification model with 5-fold cross-validation was discovered to be the most accurate method in differentiating between all kinds of benign causes of biliary obstruction. All pancreatic region malignancies (n = 19) were differentiated from benign causes (n = 75) with a sensitivity of 21.1%, specificity of 94.7% and correct rate of 80.7%.

## Discussion

Differentiating pancreatic cancer from other pathologies and healthy controls utilising analysis of VOCs from bodily fluids with FAIMS and related methods has been reported in a few studies with promising results (1–5). So far, only one study has been published on bile VOCs in which pancreatic cancer was differentiated from chronic pancreatitis (24). Our novel discovery was that pancreatic cancer can be differentiated from various malignant and benign causes of biliary obstruction by analysing bile VOCs with FAIMS.

Differentiating pancreatic cancer from both acute and chronic pancreatitis proved to work well, with high sensitivity and specificity. This is a notable finding as it has (earlier) been reported that acute and chronic pancreatitis produce VOCs somewhat similar to those in cancer (1, 2). In contrast to our study, the above-mentioned result was from analysing urine VOCs, which may explain the finding. Moreover, a broader approach to identify general characteristics differentiating between all malignancies and benign causes proved to be more challenging. This more general approach as the second aim of the study proved to be decidedly insensitive. This finding was somewhat anticipated as pathogenetic mechanisms, and presumably also VOC profiles, vary greatly between the diseases included. However, at the same time, false negatives were infrequent in differentiating between all kinds of benign causes and all kinds of malign causes of biliary obstruction.

Analysis of VOCs is based on the principle that occasionally specific molecules are expressed in different levels in different disease states. Biomarkers like this can include various tumour-specific antigens, cell-type-specific peptides and metabolic products of the tissue itself or bacteria (29, 30). We hypothesise that altered microbiome in pancreatic cancer may contribute to a distinct smell pattern detectable by FAIMS, as an altered microbiome has been noted as a risk factor in pancreatic cancer (31). Pancreatic cancer has also been associated in several studies with oral microbiota composition (32–34). The presence of microbiota in the pancreas itself and their effect on carcinogenesis is a timely research topic, as similar findings have been discovered in multiple gastrointestinal cancers (35). However, as bile passes through the pancreas, potential biomarkers for pancreatic cancer, including metabolic products of bacteria, may be more prevalent in bile than in other bodily fluids (36). On the other hand, only tumors located at the head of the pancreas potentially lead to biliary obstruction. Therefore, pancreatic juice might work even better in detecting all kinds of pancreatic disorders. In 2017 Ren et al. reported higher quantities of intratumoural bacteria in pancreatic cancer patients who developed biliary obstruction requiring biliary stenting than in patients who did not require such intervention (37). A significant increase in intrapancreatic bacterial DNA content in cancer patients has also been noted in a study by Pushalkar et al. in 2018 (38). However, intrapancreatic bacteria are known to be linked to normal, inflammatory and malignant states, and no microbiota capable of differentiating between these have so far been found (39–41).

Another probable explanation may lie in polyamine metabolism. Polyamines are strongly smelling molecules secreted into bodily fluids and working as VOCs, making them interesting biochemical markers for cancer, especially as profiles constructed of multiple biomarkers (42–46). Certain polyamines are known to be present in pancreatic cancer, but on the other hand, the same kind of polyamine profile is also related to various inflammatory states, such as pancreatitis (1). In one study a combination of urinary polyamines has recently been linked to pancreatic cancer (47). However, it is unknown which VOCs are specific to pancreatic cancer (1). In conclusion, a vast quantity of potential factors explaining our results require extensive further research, as definite factors behind VOC profile changes are yet to be explored.

The primary strength of our study is the relatively long follow-up time, which verified the diagnoses used in the statistical analysis. A fact worth mentioning is the novelty of our method, as only one study on bile VOCs has so far been published. Our results support the recent finding by Navaneethan and colleagues (24), but with longer follow-up time and by including both acute and chronic pancreatitis as differences. That study, however, used a mass spectrometer, of which an assay is capable of detecting only 22 different VOCs. In contrast to our method, FAIMS is capable of nearly exhaustive analysis of VOCs present in bile: each sample was measured in 26,112 datapoints per sample, that were further reduced to 9036 datapoints per sample to be used in statistical analysis, as explained above in Materials and methods. Moreover, mass spectrometry is a rather expensive and complicated method for routine diagnostics. FAIMS is also significantly more time efficient, as one scan and subsequent cleaning protocol take only a few minutes to complete.

The primary weakness is the small sample size. This affected the size of the pancreatic cancer group in particular. Because of the limitation, we were also unable to try to differentiate pancreatic cancer from adjacent cancers, which could have been an intriguing research question. Nevertheless, the reported results can be considered statistically significant due to the validation methods used LOO and 5-fold. This finding, however, needs to be confirmed in a more extensive setting in the future. The second weakness of our study is related to the nature of ERCP. As an invasive procedure with a risk of complications, ERCP is only performed in the presence of a definite clinical indication. This may have caused biases in study population selection. At the same time, it also constitutes a practical issue and imposes limitations on bile-based diagnostics in general.

In this study, the follow-up times were a good demonstration of the demand for this kind of supportive diagnostic method in clinical practice. This applies especially to situations where there is a known lesion in the pancreas, but its histopathological nature is unclear. In our material, working diagnoses changed from benign to malignant in 10.6% of patients in an average of three months. Many of the histopathological diagnoses were only obtained in the operation to resect the unknown lesion in the pancreatic region. After reviewing patient records retrospectively, a malignancy suggesting a FAIMS result at the time of sample collection could have improved the clinical outcomes in half of our patients diagnosed with pancreatic cancer. In one-fourth of our pancreatic cancer patients, substantial diagnostic delay (up to 14 months) or further investigations (needle biopsy, endoscopies, imaging studies) could have been avoided. The fact that a substantial proportion of patients with pancreatic cancer require ERCP for biliary stenting or decompression emphasizes the attractiveness of confirming the result and studying bile VOCs as a diagnostic aid more profoundly in the future, as patients undergoing ERCP for biliary obstruction could possibly benefit from routine quantitative analysis of bile utilising FAIMS.

We showed that patients with pancreatic cancer could be differentiated from patients with benign pancreatic lesions with a sensitivity of 100% and specificity of 77.8%. Analysing bile VOCs using FAIMS shows promising capability in the detection of pancreatic cancer and other cancers in the pancreatic area.

## Data availability statement

The raw data supporting the conclusions of this article will be made available by the authors, without undue reservation.

## Ethics statement

The studies involving human participants were reviewed and approved by Tays Expert Responsibility Area Ethics Committee. The patients/participants provided their written informed consent to participate in this study.

## Author contributions

VT was responsible for study design, general coordination, laboratory work and manuscript writing in close cooperation with SN. Significant part of the work with FAIMS device and laboratory work performed by AR and SN. AA, AS and YV worked as endoscopists gathering samples and also participated in initial study design. PK was responsible for statistical analysis. NO and JL were the professors directing and guiding whole research project. All authors contributed to the article and approved the submitted version.

## Funding

This work was supported by State Research Funding (VTR), Finland, and the Sigrid Juselius Foundation, Finland. They have no involvement in the study design, data collection, data analysis, manuscript preparation or publication decisions. The FAIMS device used is owned by NO’s study group and we were permitted to use it gratuitously.

## Acknowledgments

We thank Pekka Kumpulainen for the statistical analyses, research assistants Estefania Alvarez, Laura Matikka and Katriina Hietanen and research coordinator Satu Järvinen (Tampere University Hospital) for archive management and Virginia Mattila for proofreading.

## Conflict of interest

The authors declare that the research was conducted in the absence of any commercial or financial relationships that could be construed as a potential conflict of interest.

## Publisher’s note

All claims expressed in this article are solely those of the authors and do not necessarily represent those of their affiliated organizations, or those of the publisher, the editors and the reviewers. Any product that may be evaluated in this article, or claim that may be made by its manufacturer, is not guaranteed or endorsed by the publisher.
